# Pediatric small intestine cavernous lymphangioma detected via wireless capsule endoscopy: a rare case report

**DOI:** 10.3389/fonc.2025.1594332

**Published:** 2025-08-11

**Authors:** Xiaoyu Yang, Junwen Guan, Yi Zhang

**Affiliations:** ^1^ Department of Neurosurgery, Chengdu Women’s and Children’s Central Hospital, School of Medicine, University of Electronic Science and Technology of China, Chengdu, China; ^2^ Department of Pediatric Surgery, Chengdu Women’s and Children’s Central Hospital, School of Medicine, University of Electronic Science and Technology of China, Chengdu, China; ^3^ Department of Neurosurgery, West China Hospital, Sichuan University, Chengdu, Sichuan, China

**Keywords:** cavernous lymphangioma, wireless capsule endoscopy, small intestinal, pediatric, single-incision laparoscopic

## Abstract

**Background:**

Cavernous lymphangioma represents a common lesion of the lymphatic system, yet it is rarely encountered in the small intestine. The diagnosis of small intestinal cavernous lymphangioma poses significant clinical challenges. This case report presents the optimal diagnostic and therapeutic management of pediatric small intestinal cavernous lymphangioma.

**Case description:**

A 27-month-old male presented with recurrent syncopal episodes, hematochezia, and anemia. Small intestinal abnormalities were detected via wireless capsule endoscopy. Single-port laparoscopic surgery was subsequently performed, intraoperatively revealing a 3×3×6 cm ileal lesion located 95 cm proximal to the ileocecal valve. Segmental bowel resection was completed, with postoperative histopathological confirmation of cavernous lymphangioma.

**Conclusion:**

Pediatric cavernous lymphangioma of the small intestine is an exceedingly rare clinical disease with a multifaceted diagnostic process. Wireless capsule endoscopy is strongly recommended as a pivotal diagnostic modality for small intestine cavernous lymphangioma. Upon confirmation of the intestine lesion, immediate single-port laparoscopic resection should be implemented as the therapeutic imperative.

## 1 Introduction

Lymphangioma constitutes a congenital developmental anomaly or hamartomatous lesion of the lymphatic system, originating from lymphatic endothelial cells and vascular structures. Histopathologically, it is characterized by ectatic lymphatic channels lined by attenuated endothelial cells, surrounded by collagenous stroma or smooth muscle bundles (1). These lesions predominantly occur in cervical (75%) and axillary regions (20%), with rare involvement of orbits, mediastinum, adrenal glands, kidneys, bones, omentum, gastrointestinal tract (incidence <1%), retroperitoneum, liver, and pancreas (2). We present a 27-month-old male admitted with recurrent hematochezia and anemia. Diagnosis of small intestinal cavernous lymphangioma was confirmed via wireless capsule endoscopy, followed by successful single-port laparoscopic resection. The case report aims to enhance clinical recognition of small intestinal cavernous lymphangioma and emphasize the diagnostic significance of wireless capsule endoscopy in small intestinal lesion. This case report obtains Written informed consent from the patient’s legal guardians and adheres to the SCARE criteria (3).

## 2 Case report

### 2.1 General information

The pediatric patient was admitted with a 16-month history of recurrent melena and syncopal episodes. 16 months prior to admission(11-month-old), the child developed recurrent tarry stool. A syncopal event occurred 15 months before admission(12-month-old), prompting laboratory evaluation that revealed severe anemia (hemoglobin 52 g/L), requiring transfusion of leukocyte-depleted packed red blood cells. Subsequent abdominal ultrasonography and CT demonstrated no structural abnormalities. 3 months prior to admission(24-month-old), recurrent syncope necessitated hospitalization with critical anemia (hemoglobin 49 g/L) requiring repeat transfusion. 2 days prior to admission(27-month-old), patient’s legal guardians subsequently sought etiological investigation at our institution. Obstetric history indicated an uncomplicated pregnancy with no maternal medication exposure during the periconceptional period. The child was delivered via cesarean section at 38 weeks and 2 days gestation due to intrapartum asphyxia, with a birth weight of 3.35 kg and Apgar scores of 10 at 1 and 5 minutes.

### 2.2 Physical examination

The patient exhibited poor nutritional status with a conscious and alert mental state. Pallor was noted with mild cyanosis of the lips and pale nail beds. No petechiae or ecchymoses were observed on the skin. Oropharyngeal examination revealed non-erythematous mucosa and absence of tonsillar enlargement. Pulmonary auscultation demonstrated clear breath sounds bilaterally without adventitious sounds such as rhonchi or crackles. Cardiac evaluation showed regular rhythm with strong heart sounds and no detectable murmurs. Abdominal examination disclosed a soft, non-tender abdomen without guarding or rebound tenderness. Both hepatic and splenic margins remained non-palpable upon systematic palpation.

### 2.3 Laboratory examination

Hematological analysis revealed erythrocytopenia (RBC count 2.56×10¹²/L) with significant anemia (hemoglobin 78.00 g/L, hematocrit 25.3%). Biochemical profile demonstrated hypoproteinemia (total protein 50.0 g/L) characterized by hypoalbuminemia (albumin 34.9 g/L) and hypoglobulinemia (globulin 15.1 g/L). Fecal occult blood testing showed positivity. Serum tumor markers remained within normal detection limits.

### 2.4 Endoscopy

**Figure 1:**
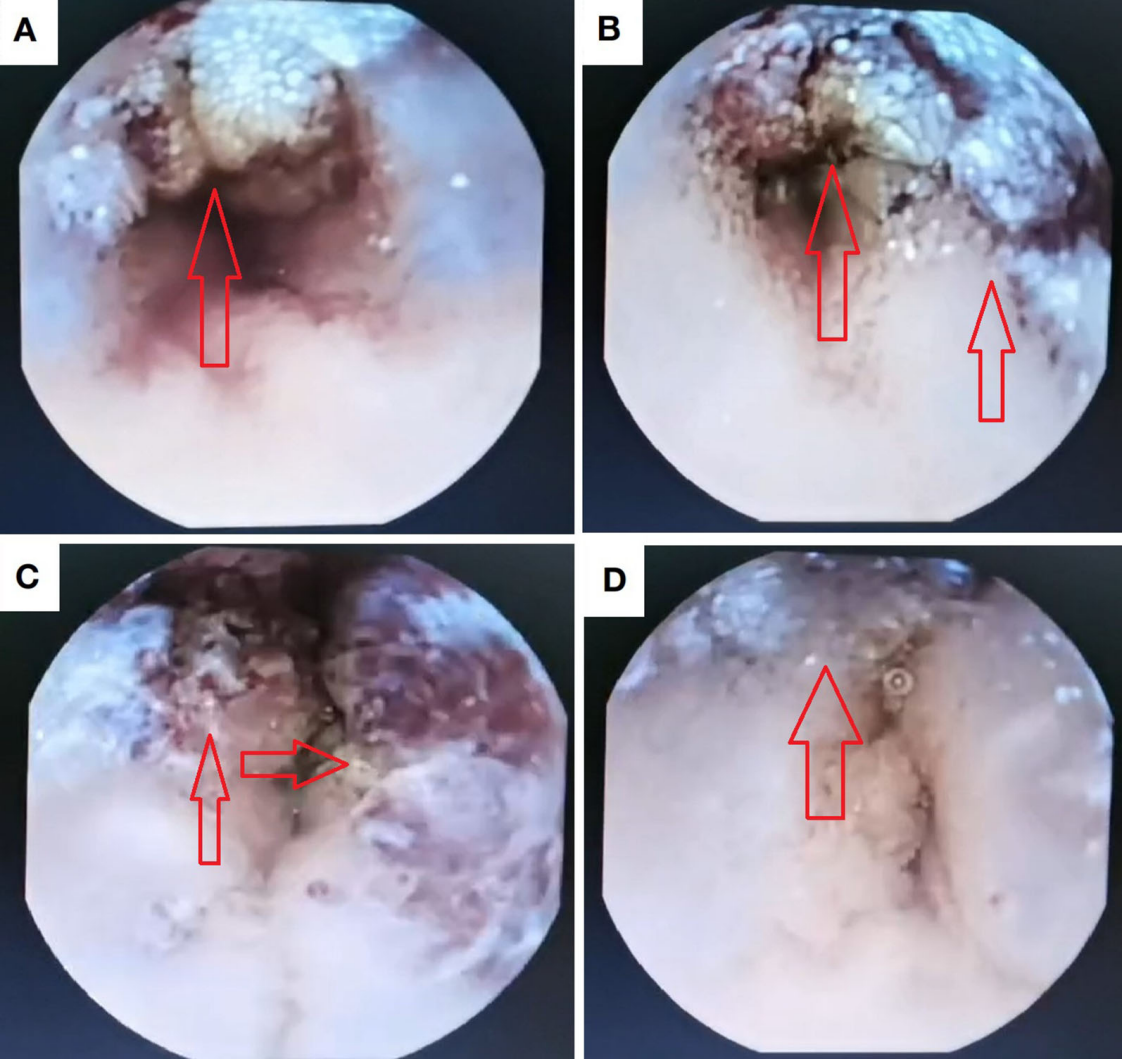
Intraluminal lesion identified via capsule endoscopy. **(a, b)** Proximal margins of the lesional bowel segment; **(c)** Intraluminal compartment within the pathological intestine; **(d)** Distal margins of the involved intestinal segment.

The patient underwent comprehensive colorectal endoscopy which demonstrated no significant abnormalities. Subsequent esophagogastroduodenoscopy (EGD) revealed unremarkable findings, with successful deployment of a wireless capsule endoscopy device in the descending duodenum. At the 4-hour post-insertion interval, capsule imaging identified a gray-brown irregular mass along the ileal wall (**Figure 1**). The device successfully traversed the ileocecal valve 5 hours after deployment.

### 2.5 Imaging examination

**Figure 2:**
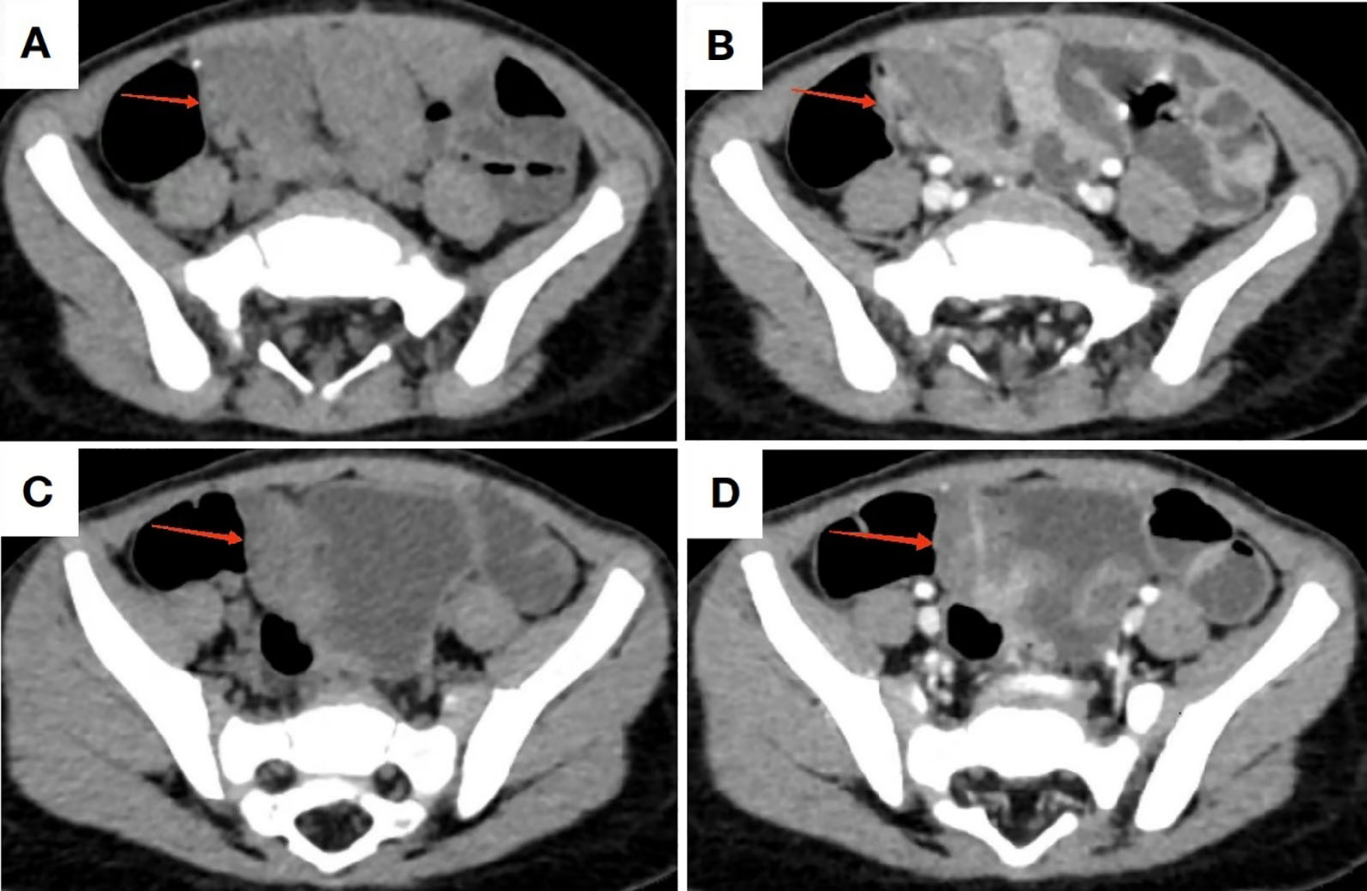
CT imaging of the lesion. **(A, C)** Non-contrast CT demonstrates bowel wall thickening in the distal ileum with minimal calcifications; **(B, D)** Contrast-enhanced CT reveals mild to moderate enhancement of the pathological segment.

Abdominal ultrasonography(US) revealed no significant abnormalities. Technetium-99m scintigraphy demonstrated absence of abnormal radiotracer accumulation in the abdominal region. Contrast-enhanced CT imaging identified a 65-mm segmental circumferential mural thickening in the distal ileum, located superior to the right aspect of the urinary bladder. The thickened bowel wall measured up to 10 mm in maximal cross-sectional diameter, demonstrating a precontrast attenuation value of 35 Hounsfield units (HU) with punctate calcification and mild enhancement post-contrast administration. Luminal narrowing was noted without proximal bowel dilatation. Perienteric fat planes remained preserved (**Figure 2**).

### 2.6 Surgical procedure

**Figure 3:**
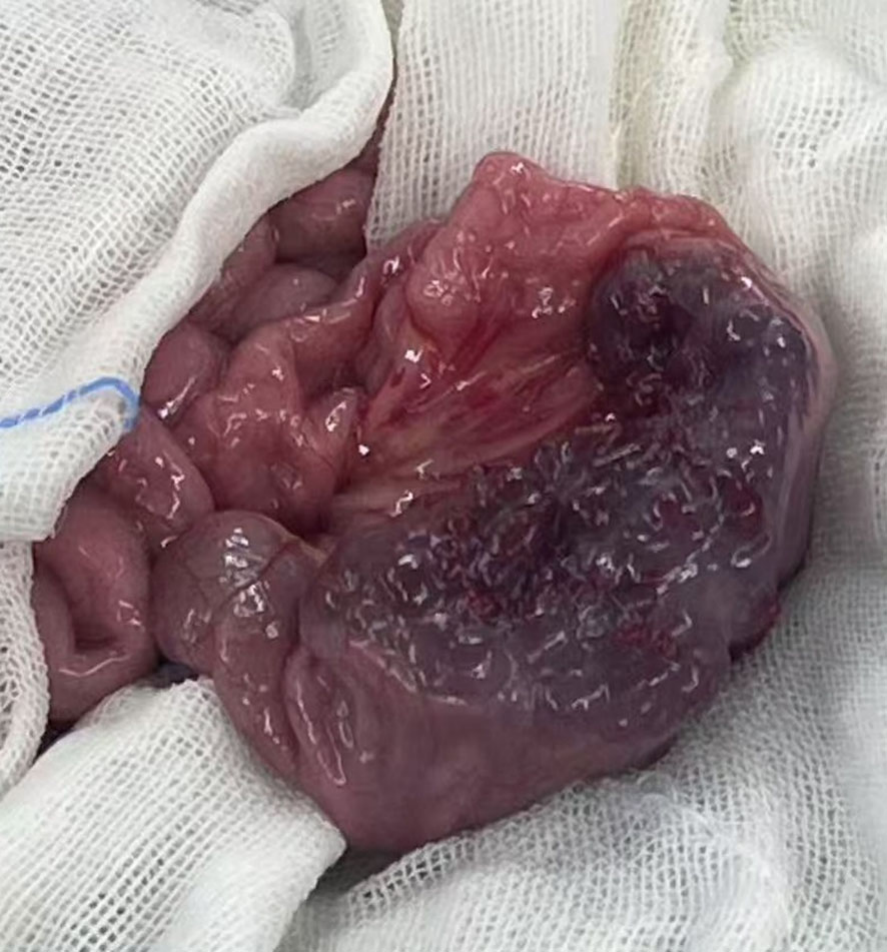
Intraoperative identification of cavernous lymphangioma involving the small intestine.

Based on the patient’s clinical history, physical examination, and ancillary investigations, a provisional diagnosis of small intestinal neoplasm was established, prompting single-port laparoscopic surgical intervention. Following successful anesthesia induction, the patient was positioned in the supine position with standard antiseptic preparation and draping. A 4-cm vertical transumbilical incision was created, through which a disposable wound retractor fixation system was deployed. The single-port laparoscopic platform and articulated laparoscopic instruments were prepared. Intraoperative exploration identified a gray-brown irregular mass (3×3×6 cm) involving the ileal wall 95 cm proximal to the ileocecal junction. We exteriorized it through the single-port incision (**Figure 3**). After meticulous dissection and ligation of the mesenteric vasculature supplying the affected bowel segment, the surgical team resected the affected bowel segment with 3 cm macroscopic margins proximal and distal to the lesion. End-to-end intestinal anastomosis was performed using interrupted sutures under tension-free conditions. Following confirmation of satisfactory anastomotic integrity, the bowel segment was carefully reintroduced into the abdominal cavity. Prior to closure, systematic reinspection confirmed absence of intestinal torsion or active hemorrhage. The wound retractor system was removed, and the fascial layers were closed with absorbable sutures.

### 2.7 Pathological findings

**Figure 4:**
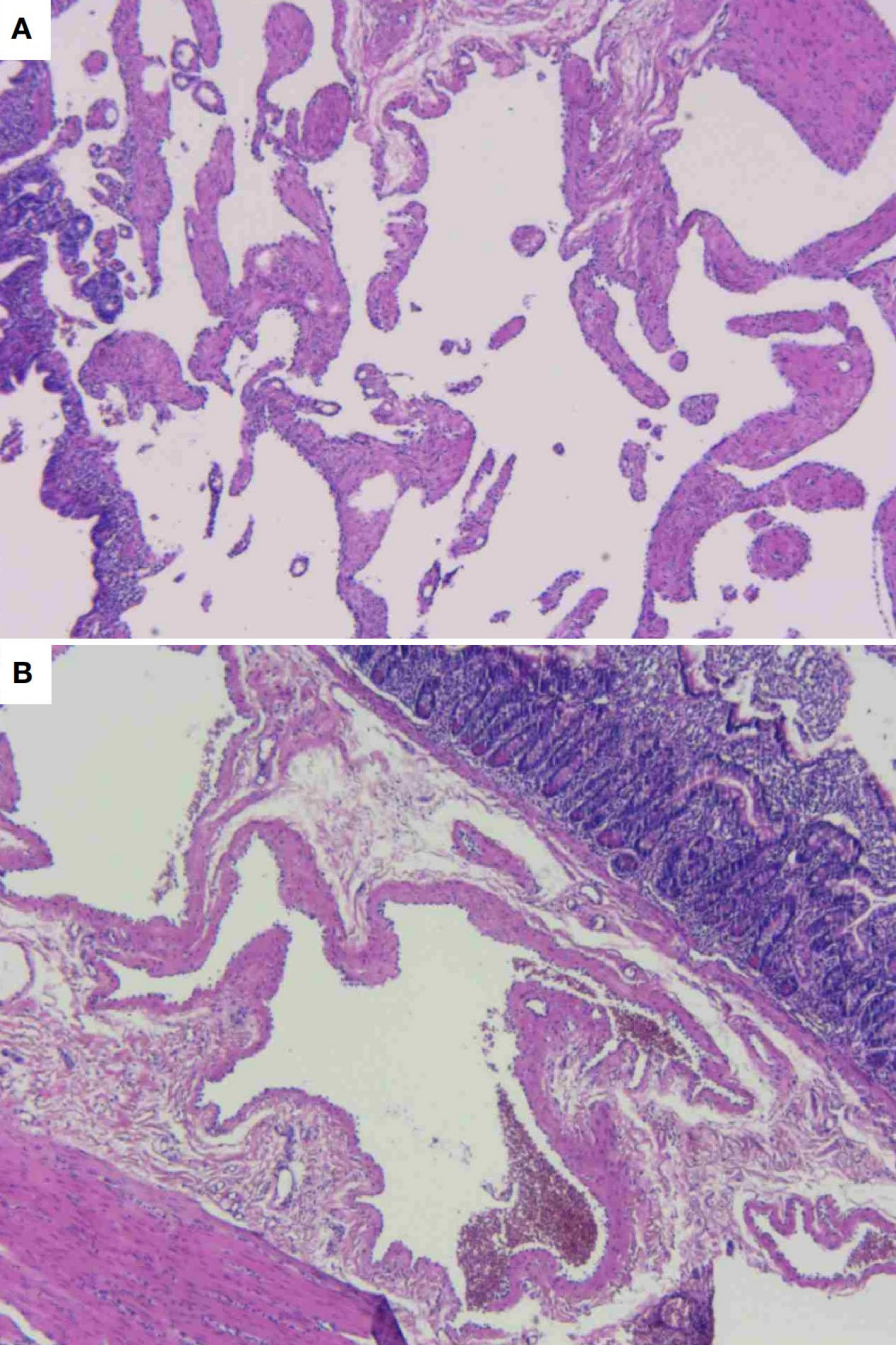
Histopathological photomicrograph of the lesion. **(A)** Dilated lymphatic channels exhibiting attenuated endothelial cells adherent to the vessel wall. **(B)** Dilated lymphatic channels in the mucosal and submucosal layers of the intestine, with associated muscular tissue in proximity.

Gross examination revealed a gray-brown slightly elevated lesion (4.5×2.5 cm) on the mucosal surface. The serosal aspect demonstrated an irregular gray-brown mass measuring 3×3×6 cm, exhibiting a spongy cut surface with reddish-brown fluid accumulation. Histopathological examination showed dilated lymphatic channels within the intestinal mucosa and adjacent tissues, lined by attenuated endothelial cells (**Figure 4**). Immunohistochemical staining demonstrated positive immunoreactivity for D2-40. Pathological Diagnosis: Cavernous lymphangioma of the small intestine (WHO Classification of Soft Tissue Tumors, 2020).

### 2.8 Postoperative follow-up

**Figure 5:**
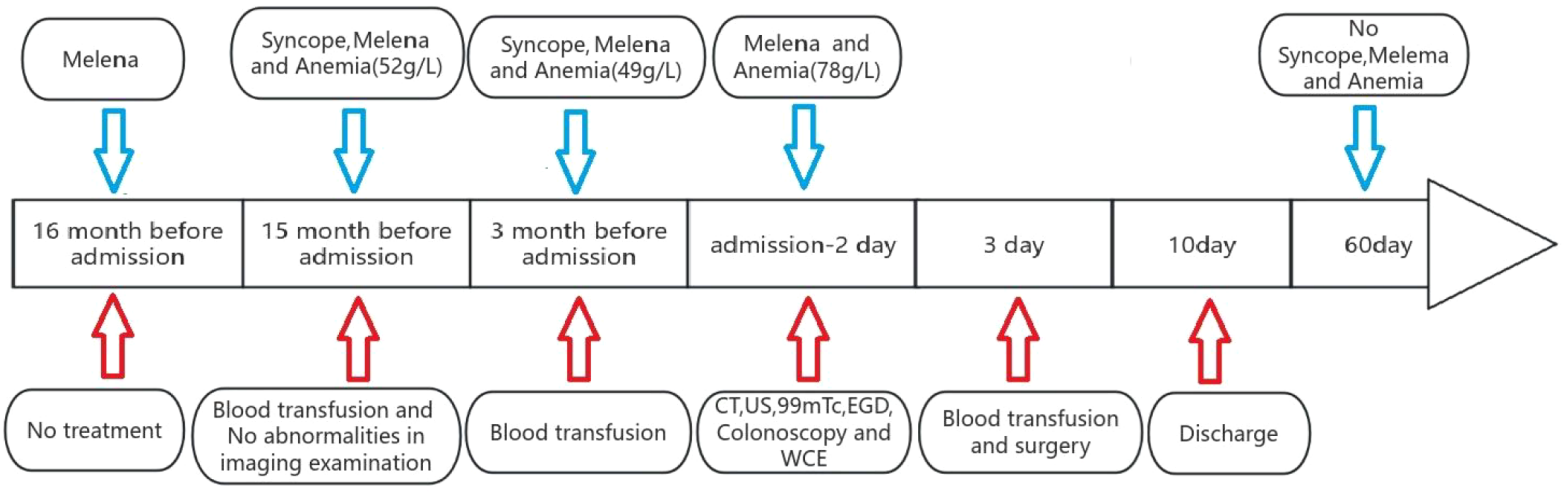
The complete disease progression timeline in the pediatric case.

The patient underwent scheduled follow-up assessments for two months postoperatively. Serial evaluations revealed normalization of dietary intake and bowel movements, with complete resolution of hematochezia. Serial hemoglobin measurements remained within normal physiological limits, demonstrating no significant declining trend (**Figure 5**).

## 3 Discussion

Lymphangioma is a malformation characterized by abnormal development of lymphatic system structures, which can be histopathologically classified into capillary lymphangioma, cavernous lymphangioma, and cystic lymphangioma (4). These lesions predominantly occur in the head and neck region (75%) and axilla (20%), with rare involvement of the gastrointestinal tract (<1%) (2) Typical clinical manifestations of small intestinal cavernous lymphangioma include hematochezia, intestinal obstruction, and abdominal pain (5). In this case report, the cavernous lymphangioma was localized to the ileum, 95 cm proximal to the ileocecal valve. Conventional diagnostic modalities, including esophagogastroduodenoscopy (EGD) and colonoscopy, failed to visualize the lesion. Additionally, cross-sectional imaging studies did not provide definitive guidance for clinical decision-making. However, wireless capsule endoscopy played a pivotal role in establishing the diagnosis and guiding subsequent management.

Prior to the development of wireless capsule endoscopy (WCE), evaluation of the small intestine was restricted to invasive procedures (e.g., intraoperative enteroscopy) or suboptimal diagnostic methods such as contrast radiography (6). Wireless capsule endoscopy revolutionized small bowel endoscopy by providing mucosal visualization with noninvasive method (7). Wireless capsule endoscopy is an advanced medical technology that employs a wireless, capsule-sized miniature camera to perform noninvasive visualization and diagnostic assessment of the gastrointestinal tract (8). Originally designed for evaluating small bowel pathologies, this innovation addresses the inherent limitations of conventional endoscopy, which cannot reliably access the entirety of the small intestine (9). The capsule progresses passively through the digestive tract via physiological peristalsis, capturing images at a rate of several frames per second via an integrated miniature camera (10). These images are transmitted wirelessly to an external recording device for subsequent clinician interpretation. Wireless capsule endoscopy is strongly recommended as a effective diagnostic method for patients of recurrent gastrointestinal hemorrhage that remain undiagnosed following conventional ancillary investigations (11).

The management of lymphangioma is determined by factors including anatomic location, tumor dimensions, and invasiveness (12). Surgical resection of the affected bowel segment remains the first-line therapeutic approach for intestinal lymphangioma (13). Given the benign nature of lymphangiomas and their absence of distant metastasis to tissues or organs, localized excision of the diseased bowel typically achieves favorable long-term prognosis (13). Contemporary surgical practice has shifted toward minimally invasive and precision-oriented techniques, with both surgeons and patients sharing a priority of minimizing surgical scarring. The umbilicus has gained attention as an optimal site for scar-concealed incisions (14). While not the first reported case globally, cavernous lymphangioma predominantly occurs in the head and neck region, with ileal wall involvement representing an exceptionally rare presentation (**Table 1**). This case exemplifies an exemplary management paradigm by utilizing capsule endoscopy for diagnosis and single-port laparoscopy as the minimally invasive surgical modality, constituting a reference-worthy diagnostic and therapeutic framework for such rare entities. In the present case, we employed a single-incision laparoscopic exploration to localize the lesion within the small intestine, followed by ex vivo resection of the lymphangioma via a transumbilical approach. This method represents a synthesis of conventional surgical principles and laparoscopic advantages, optimizing both surgery accuracy and cosmetic outcomes.

**Table 1:** English literature on PubMed of small intestine cavernous lymphangioma.

Ref	Year	Age/sex	Symptom	Location	Tumor size(cm)	Diagnostic method	Management
Davis et al (15)	1987	53/F	substernal burning and pain	Second portion of duodenum	2.5	Upper gastrointestinal series	Endoscopyresection
Hanagiri et al (16)	1992	53/M	abdominal pain	250 cm distal to Treitz	2.5*2.0	selective intestinograms	Laparotomy resection
Morris et al (17)	2011	34/F	dyspnoea	proximal jejunum	5.3*4*1.5	capsule endoscopy	Laparotomy resection
Zhao et al (18)	2022	32/F	abdominal pain and vomiting	15 cm distal to Treitz	9*5*2	Contrast-enhanced CT	Laparotomy resection
Liu et al (19)	2024	66/M	melena and fatigue	200 cm distal to Treitz	10	capsule endoscopy	Laparotomy resection

## 4 Conclusions

In summary, small intestinal lymphangioma represents a rare etiology of gastrointestinal hemorrhage, frequently underdiagnosed due to limitations of conventional diagnostic modalities. Wireless capsule endoscopy is recommended as the first-line diagnostic method for small intestinal lymphangioma, though its cost-prohibitive nature often delays implementation.

Given the propensity of intestinal lymphangiomas to cause persistent hemorrhage, complete surgical excision provides the only pathway to definitive resolution. Therefore, expedited surgical intervention should be pursued upon clinical suspicion, in accordance with emergency surgery protocols.

## Patient's perspective

The child’s parents stated that they had sought medical attention at six different hospitals prior to this consultation, undergoing both colonoscopy and gastroscopy without lesion detection. Their child received three blood transfusions totaling five therapeutic units. The parents expressed profound gratitude for the capsule endoscopy that successfully identified the lesion, and the subsequent single-port laparoscopic resection which they described as a “therapeutic milestone”.

## Data Availability

The original contributions presented in the study are included in the article/**Supplementary Material**. Further inquiries can be directed to the corresponding author.
